# N_2_ as an Efficient IR Probe Molecule for the Investigation of Ceria-Containing Materials

**DOI:** 10.3390/molecules29153608

**Published:** 2024-07-30

**Authors:** Kristina K. Chakarova, Mihail Y. Mihaylov, Bayan S. Karapenchev, Iskra Z. Koleva, Georgi N. Vayssilov, Hristiyan A. Aleksandrov, Konstantin I. Hadjiivanov

**Affiliations:** 1 Institute of General and Inorganic Chemistry, Bulgarian Academy of Sciences, 1113 Sofia, Bulgaria; bkarapench@uni-sofia.bg (B.S.K.); haa@chem.uni-sofia.bg (H.A.A.); kih@svr.igic.bas.bg (K.I.H.); 2 Faculty of Chemistry and Pharmacy, University of Sofia, 1126 Sofia, Bulgaria; ohik@chem.uni-sofia.bg (I.Z.K.); gnv@chem.uni-sofia.bg (G.N.V.)

**Keywords:** adsorption, ceria, dinitrogen, FTIR spectroscopy, probe molecules

## Abstract

Ceria and ceria-based catalysts are very important in redox and acid-base catalysis. Nanoceria have also been found to be important in biomedical applications. To design efficient materials, it is necessary to thoroughly understand the surface chemistry of ceria, and one of the techniques that provides such information about the surface is the vibrational spectroscopy of probe molecules. Although the most commonly used probe is CO, it has some disadvantages when applied to ceria and ceria-based catalysts. CO can easily reduce the material, forming carbonate-like species, and can be disproportionate, thus modifying the surface. Here, we offer a pioneering study of the adsorption of ^15^N_2_ at 100 K, demonstrating that dinitrogen can be more advantageous than CO when studying ceria-based materials. As an inert gas, N_2_ is not able to oxidize or reduce cerium cations and does not form any surface anionic species able to modify the surface. It is infrared and transparent, and thus there is no need to subtract the gas phase spectrum, something that often increases the noise level. Being a weaker base than CO, N_2_ has a negligible induction effect. By using stoichiometric nano-shaped ceria samples, we concluded that ^15^N_2_ can distinguish between surface Ce^4+^ sites on different, low index planes; with cations on the {110} facets and on some of the edges, Ce^4+^−^15^N_2_ species with IR bands at 2258–2257 cm^−1^ are formed. Bridging species, where one of the N atoms from the molecule interacts with two Ce^4+^ cations, are formed on the {100} facets (2253–2252 cm^−1^), while the interaction with the {111} facets is very weak and does not lead to the formation of measurable amounts of complexes. All species are formed by electrostatic interaction and disappear during evacuation at 100 K. In addition, N_2_ provides more accurate information than CO on the acidity of the different OH groups because it does not change the binding mode of the hydroxyls.

## 1. Introduction

Materials based on ceria are highly effective for a wide range of applications, such as heterogeneous catalysis, chemical sensing, and biomedicine. In particular, in catalysis, ceria plays a key role as an active support or component in various processes for automotive exhaust gas aftertreatment, the oxidation of volatile organic compounds, CO_2_ conversion, low-temperature water-gas shift (WGS) reaction, organic compound synthesis, and more [1,2,3,4].

The importance of ceria for these applications stems from its exceptional surface (and partly bulk) properties, mainly related to the ability of Ce ions to easily switch between Ce^4+^ and Ce^3+^ oxidation states [1,3]. However, some organic reactions, such as dehydration and ketonization, are catalyzed by acid-base sites, while others, such as addition, substitution, isomerization, and ring-opening reactions, require both acid-base and redox centers [5].

Due to its great significance, CeO_2_ has been highly studied, and the development of active materials based on CeO_2_ represents a key current topic in science [1,2,3,4,5,6,7,8,9,10,11,12,13,14,15,16,17]. A search in Scopus (keywords: ceria or cerium (di)oxide and surface) shows a sharp increase in interest in this field since the beginning of the century: 548 documents in 2000 and 16,800 documents in 2023.

Stoichiometric CeO_2_ possesses a cubic fluorite structure with eightfold coordination for cerium ions and tetrahedral coordination for oxygen anions. According to the large size of the cations, CeO_2_ shows medium Lewis acidity and relevant surface basicity. Under reducing conditions, it releases oxygen, creating oxygen vacancies within the preserved fluorite structure, and in an oxidizing atmosphere, it is easily oxidized back to CeO_2_ [6,7]. Note that the creation of oxygen vacancies can modify the acid-base properties of ceria.

The surface properties of CeO_2_ depend on the size and shape of the crystallites [8,9,10]. Smaller particles are characterized by a larger active surface area, more edges, steps, and vertices, and favor reduction. Recently, to clarify the relationship between activity and structure, special attention has been given to ceria nanoparticles with specific shapes [11,12,13,14,15,16,17], as they preferentially expose certain low-index planes. Thus, ideal nanocubes are enclosed by the {100} facets, while ideal nanooctahedra are enclosed by the {111} facets. It has been found that, compared to the {111} facet, the {100} and {110} facets are more active in CO oxidation [10,12,18,19,20,21], H_2_ oxidation, WGS [8,22], and the reverse WGS reaction [23]. It has also been reported that the {100} facet is more active in the dehydrogenation of 2-propanol to acetone, while the {111} facet demonstrated higher activity in propanol dehydration [24].

The design of effective materials based on CeO_2_ requires a deep understanding of the surface chemistry of CeO_2_, and one of the techniques that provides rich information about the surface is vibrational spectroscopy [25,26]. This technique can directly observe surface hydroxyl groups and some impurities, such as residual carbonates or nitrates. Specifically, for cerium dioxide and materials based on cerium dioxide, vibrational spectroscopy can also be used to monitor its degree of reduction through the Ce^3+^ spin-orbital ^2^F_5/2_ → ^2^F_7/2_ electronic transition at around 2147–2110 cm^−1^ [6,7,27,28,29,30,31,32].

Additional data on the nature of various surface sites can be obtained using probe molecules. Among the different probes, CO is the most informative and widely used [25]. However, the use of CO to characterize CeO_2_-based materials has some issues. CO can easily reduce CeO_2_, forming carbonates, and can even be disproportionate, thus modifying the surface [33]. Nonetheless, reactive adsorption can be largely suppressed at low temperatures. When forming carbonyls with Ce^4+^ cations, CO is bound by σ and electrostatic bonds, which leads to a blue shift of ν(C−O). There is no consensus in the literature regarding the assignment of different carbonyl bands, especially the oxidation state of cerium in the complexes. The acidity of the hydroxyl groups is also under debate.

Recently, we published a comprehensive study on CO adsorption on stoichiometric CeO_2_, combining IR spectroscopy and DFT calculations [34]. It was revealed that ν(C−O) is sensitive to the localization of the Ce^4+^ adsorption sites and decreases in the order edge/apex, {110}, {100}, and {111} facets. However, significant band shifts with increasing coverage, due to the inductive effect, the formation of dicarbonyls, and associated bridging carbonyls, hinder a quantitative assessment of these centers. Additionally, the results indicated that CO adsorption could induce reconstructions in some hydroxyl groups, thus compromising the measurement of proton acidity.

In search of a more appropriate probe molecule, we selected dinitrogen. Although ν(N−N) of dinitrogen is not IR-active in the gas phase, it becomes activated upon adsorption due to symmetry lowering. ^15^N_2_ was chosen in order to avoid any hindrance from gaseous CO_2_. The use of N_2_ as a probe molecule to test surface acidity at low temperatures has been proposed for different systems [35,36,37,38,39,40,41,42,43]. However, to the best of our knowledge, dinitrogen has not yet been applied as a probe for CeO_2_-based systems. Compared to CO, dinitrogen offers certain advantages. There is no need to subtract the spectrum of the gas phase. As an inert gas, N_2_ cannot oxidize or reduce cerium cations, and it does not form any surface anionic species capable of modifying the surface. Being a weaker base than CO, N_2_ is not expected to have a noticeable induction effect or cause surface reconstructions.

Preliminary studies have also indicated that dinitrogen is also a suitable probe for detecting Ce^3+^ sites. Investigations on this subject are now in progress and will allow for a comprehensive understanding of the surface chemistry of ceria.

In this work, we demonstrate the potential of dinitrogen as an IR probe molecule for studying stoichiometric ceria nanoparticles. Dinitrogen adsorption measurements were conducted in parallel with those of CO, allowing for direct comparison. The interpretation of the experimental data was also supported by theoretical modeling of the possible dinitrogen complexes and by calculating their wavenumber and binding energy.

## 2. Results and Discussion

### 2.1. Basic Characteristics of the Samples

In this work, we studied three ceria samples: nanocubes (CeO_2_-NC), nanopolyhedra (CeO_2_-NP), and nanorods (CeO_2_-NR). The different shapes of the crystallites are achieved by varying the conditions of the hydrothermal synthesis, the concentration of NaOH, and the reaction temperature. The main characteristics of these samples are reported elsewhere [34], and the most important are summarized in Table 1. Energy dispersive X-ray (EDX) analysis did not show the presence of residual sodium [34].

Briefly, the CeO_2_-NC sample consisted of particles mainly enclosed by the {100} facets, although some {110} and {111} facets were exposed to a lesser extent. The CeO_2_-NP sample was obtained by calcination of CeO_2_-NC at 923 K. In agreement with earlier reports [34,44,45], this treatment led to a strong increase in the relative exposure of the {111} facets at the expense of the {100} facets. The CeO_2_-NR sample exposed mainly the {110} facets, followed by {111} and {100}. It was characterized by the smallest particles and had the highest specific surface area. The main particle size of CeO_2_-NC was 27 nm, and the surface area was 31 m^2^ g^−1^. These values only slightly decreased after calcination at 923 K to produce CeO_2_-NP.

Therefore, depending on the shape of the crystallites, different low-index facets predominated in each of our samples. The models of the different facets we used are described below. In all cases, the nanoparticles have a significant number of centers located at edges, corners, and defects.

### 2.2. Lewis Acidic Ce^4+^ Sites

Before the adsorption of probe molecules, the samples were subjected to activation consisting of successive thermal treatments in O_2_ and a vacuum. This process aimed to remove water, organic impurities, carbonates, etc., from the surface and to ensure the stoichiometric state of the cerium samples. After activation, virtually all the cerium is in the Ce^4+^ form [46], as evidenced by the absence of the Ce^3+^ electronic transition band at approximately 2115 cm^−1^ [6,19], the yellow color [47], and the lack of the formation of reactive oxygen species (O_2_^−^, O_2_^2−^) upon O_2_ adsorption [34,46]. However, small amounts of oxyhydroxide and carbonates remain. Subsequently, the samples were brought into contact with heavy water and gradually dehydrated and dehydroxylated by evacuation at elevated temperatures to create bare Ce^4+^ Lewis acid sites.

**Figure 1:**
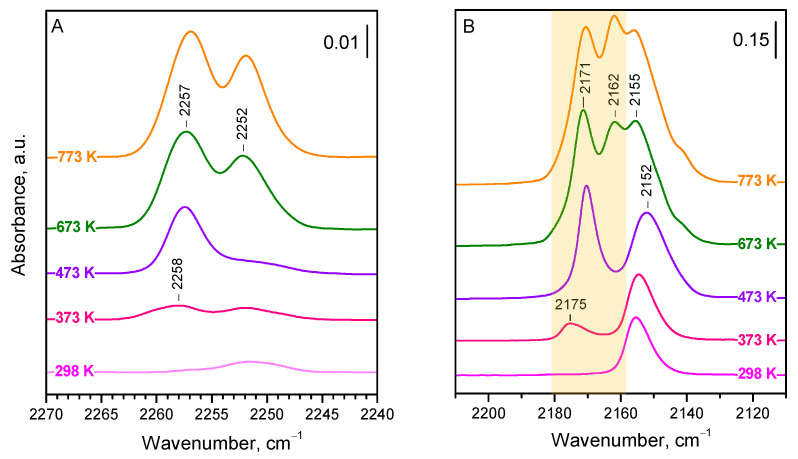
FTIR spectra of ^15^N_2_ and CO adsorbed at 100 K on CeO_2_-NC pre-evacuated at different temperatures, as marked on the spectra. (**A**) Adsorbed ^15^N_2_ at maximum coverage (10 mbar equilibrium pressure). (**B**) Adsorbed CO at intermediate coverage (for details, see text). The region of the carbonyl bands corresponding to ^15^N_2_ bands is highlighted.

Adsorption of ^15^N_2_ was first studied on the CeO_2_-NC sample evacuated at different temperatures (Figure 1A). An increase in evacuation temperature leads to gradual sample dehydroxylation and the creation of bare Ce^4+^ Lewis acid sites [34].

The introduction of ^15^N_2_ (10 mbar) to the sample evacuated at 298 K leads only to the appearance of a very weak band at 2252 cm^−1^, which is attributed to OH−^15^N_2_ interaction [36,40,41]. Indeed, the band develops alongside the shifting of hydroxyl bands (for more details, see below). The situation is similar with the sample evacuated at 373 K, but, in this case, another weak band at 2258 cm^−1^ is discernible. This later band significantly increases in intensity after preliminary evacuation at 473 K, while the OH−^15^N_2_ band fades. When the sample was evacuated at 673 K, the band at 2257 cm^−1^ further developed, and a strong band at 2252 cm^−1^ appeared. Both bands were even slightly more intense in a sample evacuated at 773 K.

The results described show that the spectra of dinitrogen adsorbed on stoichiometric CeO_2_-NC are relatively simple and consist of two bands (at 2257 and 2252 cm^−1^) the intensity of which strongly depends on the sample dehydroxylation degree. In fact, there are two species with different stabilities associated with the 2252 cm^−1^ band. The band at 2257 cm^−1^ seems to have a component at 2258 cm^−1^. We noted that the band maxima practically do not depend on the coverage (see Figure S1), which suggests (i) the lack of lateral and vibrational interaction between the adsorbed molecules and (ii) no measurable formation of geminal species. In any case, the results are somewhat surprising, because the use of CO as a probe molecule has clearly indicated the existence of at least three kinds of Ce^4+^ sites with different acidities [34]. In order to correctly assign the bands, we compared the results with results on CO adsorption. As already noted, the CO adsorption experiments were performed immediately after the experiments on N_2_ adsorption.

Now, we consider the results obtained with CO as a probe (Figure 1B), which are consistent with our previous report [34]. Because CO is more strongly adsorbed than ^15^N_2_ and, at high coverage, forms geminal structures, we examined the spectra registered at intermediate (ca. 50%) coverage. There are three main bands in the spectra. The first band is at 2175–2171 cm^−1^ and it first appears with the sample evacuated at 373 K, reaching maximal intensity with the sample evacuated at 473 K. This band is attributed to CO adsorbed on Ce^4+^ sites from the {110} facets (component at 2171 cm^−1^) and Ce^4+^ on edges (component at 2175 cm^−1^) [34]. The second band is at 2162 cm^−1^ and it becomes well discernible with the sample evacuated at 673 K. This band is associated with the {100} facets, which retain OH groups at relatively high temperatures. Finally, the third band is at ca. 2155 cm^−1^ and is of significant intensity even with the sample evacuated at ambient temperature. This band was attributed to CO adsorbed on the {111} facets, which are not hydroxylated and hold only weakly adsorbed water molecules at ambient conditions.

Comparison between the spectra of adsorbed ^15^N_2_ (Figure 1A) and CO (Figure 1B) allows for the determination of the following conclusions:

- Dinitrogen, unlike CO, does not bind to the weakly acidic surface Ce^4+^ sites located on the {111} facets. This follows from the fact that there is no ^15^N_2_ band in Figure 1A changing in concert with the CO band at 2155–2152 cm^−1^ (Figure 1B).
- ^15^N_2_ adsorbed on the {110} facets and some edges is characterized by a band at 2258–2257 cm^−1^. Indeed, this band follows the changes of the CO band at 2175–2171 cm^−1^. We tentatively assign the component at 2158 cm^−1^ to dinitrogen on the edges and the component at 2257 cm^−1^ to dinitrogen on the {110} facets.
- The band at 2252 cm^−1^ (2253 cm^−1^ at low coverage) is attributed to ^15^N_2_ on the {100} facets. It gains significant intensity when the sample is evacuated at 673 K or above, exactly the same as the carbonyl band at 2162 cm^−1^. A minor component of this band, easily disappearing after evacuation, characterizes OH−^15^N_2_ adducts.

**Figure 2:**
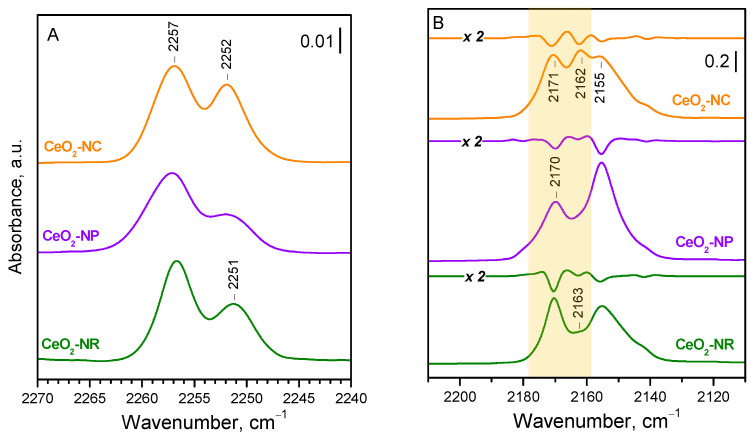
FTIR spectra of ^15^N_2_ and CO adsorbed at 100 K on differently shaped ceria nanoparticles pre-evacuated at 773 K. (**A**) Adsorbed ^15^N_2_ at maximum coverage (10 mbar equilibrium pressure). (**B**) Adsorbed CO at intermediate coverage (for details, see text). The region of the carbonyl bands corresponding to the ^15^N_2_ bands is highlighted. The corresponding second derivative is shown above the spectrum.

To obtain further confirmation of the proposed assignments, we compared the spectra of ^15^N_2_ adsorbed on three samples, CeO_2_-NC, CeO_2_-NP, and CeO_2_-NR, which differ in their exposed faces (Figure 2A). To ensure a high dehydroxylation degree, the samples were evacuated at 773 K. As expected, the band at 2252 cm^−1^, due to the dinitrogen adsorbed on {100} facets, is most intense with CeO_2_-NC, and weak with the CeO_2_-NP sample, consistent with the development of the inert {111} at the expense of the {100} facets. The band at 2257 cm^−1^ is most intense with the CeO_2_-NR sample, in agreement with the high exposure of the {110} facets. These observations are in full agreement with the results on CO adsorption (Figure 2B).

Although the general picture of ^15^N_2_ adsorption on ceria seems to be clear, some additional details can be extracted from the analysis of the spectra recorded at decreasing coverage (Figure S1). The maximum of the band at 2257 cm^−1^ appears to be practically coverage-independent. However, a slight blue shift, less than 1 cm^−1^, is observed for the band at 2252 cm^−1^ with coverage decreasing. This shift can be justified by the fact that, at high coverage, the band is superimposed on the band of the OH−^15^N_2_ complexes at ca. 2252 cm^−1^. However, it seems that a negligible shift also occurs with the component due to ^15^N_2_ polarized by Ce^4+^ sites. In any case, the wavenumber of the band at intermediate coverages, after destroying the OH−^15^N_2_ species, is close to 2253 cm^−1^.

### 2.3. DFT Modeling of N_2_ Adsorption on Ceria Models

**Figure 3:**
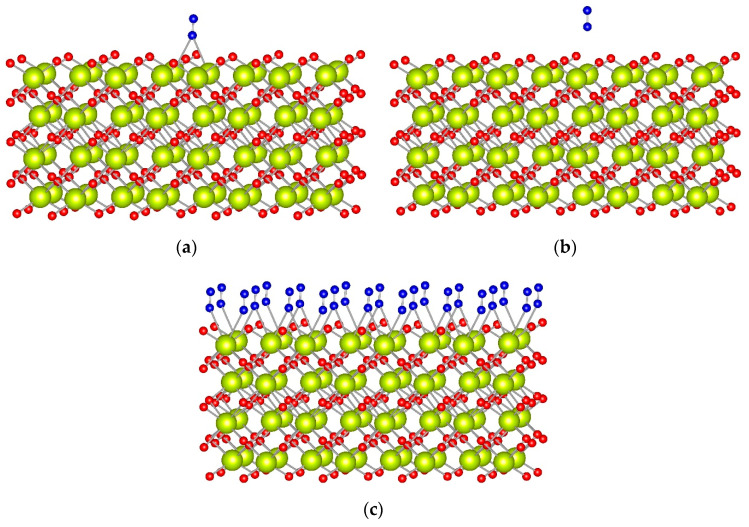
Optimized complexes of an N_2_ molecule in (**a**) bridge and (**b**) top coordination to the CeO_2_(100) surface model. (**c**) A full monolayer of N_2_ molecules adsorbed in bridge coordination to the CeO_2_(100) surface model. CeO_2_(100) model was created with surface O centers moved along the “100” direction. Color coding: Ce—yellow, O—red, and N—blue.

**Figure 4:**
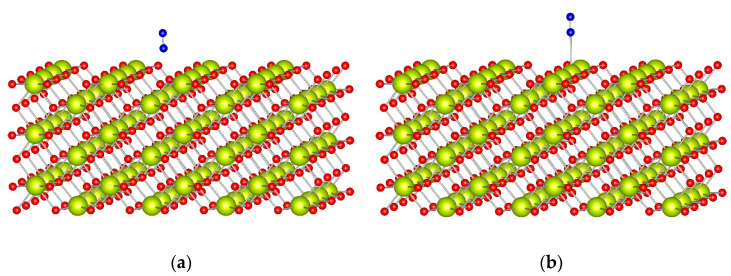
Optimized complexes of an N_2_ molecule in (**a**) bridge and (**b**) top coordination to the CeO_2_(110) surface model. Color coding: Ce—yellow, O—red, and N—blue.

We theoretically investigated the adsorption of N_2_ on Ce^4+^ cations located on the surface of four ceria models: the CeO_2_(111) surface model with step, the ideal CeO_2_(100) and CeO_2_(110) surface models, as well as the Ce_40_O_80_ nanoparticle (see Table 2). The obtained structures for dinitrogen adsorption on CeO_2_(100) and CeO_2_(110) surfaces are shown in Figure 3 and Figure 4, respectively. For direct comparison with experimental values, all frequencies in the text correspond to ^15^N_2_.

When N_2_ is adsorbed to a four-coordinated Ce^4+^ cation located at the corner of the Ce_40_O_80_ nanoparticle model, its binding energy (BE) is −0.30 eV, while the Ce^4+^−N distance is 309 pm. The calculated N−N vibrational wavenumber, 2254 cm^−1^, is only 2 cm^−1^ higher than the corresponding calculated value for the gas phase ^15^N_2_ molecule, at 2252 cm^−1^. The BE per N_2_ ligand for the Ce^4+^(N_2_)_2_ complex, −0.27 eV, is slightly lower than the value for the monoligand complex. Similarities between both complexes were also observed for the Ce^4+^−N distances, which are only 1–2 pm longer in the diligand complex than in the monoligand complex. The calculated frequencies for the Ce^4+^(N_2_)_2_ complex are 2258 and 2254 cm^−1^.

The binding energy of N_2_ is −0.26 ÷ −0.27 eV when the ligand is adsorbed at a top position to a Ce^4+^ cation located at the terrace of the CeO_2_(111) surface (Figure S2). The calculated N−N vibrational wavenumber, 2255 cm^−1^, is slightly shifted by 2–3 cm^−1^ to higher frequencies with respect to the corresponding value for the ^15^N_2_ in the gas phase, at 2252 cm^−1^. Most probably, the low binding energy of N_2_ is the reason that we do not detect an experimental band when N_2_ is adsorbed to CeO_2_(111). Stronger adsorption was found when N_2_ interacts with the low-coordinated Ce^4+^ cation at the edge of the step, as the BE value is −0.31 eV, while the calculated N−N wavenumber is 2261 cm^−1^.

As recently reported, the CeO_2_(100) surface is fully hydroxylated at ambient conditions [34,48], and, upon dehydroxylation, half of the oxygen anions are removed as part of the water molecules [34]. For this reason, for this surface, we created two models where we moved half of the surface O to the other (initially cerium-terminated) surface. In the first model, the surface O centers were moved along the <100> direction. In the second, oxygen atoms were moved in checkerboard style. Both models provide very similar results, as the N_2_ prefers to interact with two Ce^4+^ cations in a bridge coordination (Figure 3a and Figure S3). In this position, the BE of N_2_ is ~−0.45 eV, which is twice as large as an absolute value as the BE of N_2_ at top coordination, at −0.20 eV. Despite the different stabilities, the N−N vibrational wavenumber is essentially the same in both coordination modes, 2253 cm^−1^, and does not change much from the corresponding value for the ^15^N_2_ molecule in the gas phase.

We also calculated a full monolayer of N_2_ molecules initially located in the bridge or top positions. The most stable structure is again when all N_2_ molecules are in bridge positions (Figure 3c), as the binding energy per N_2_ molecule, −0.43 eV, is the same as the corresponding value for the structure with only one N_2_ adsorbate, manifesting the lack of significant lateral interactions between the adsorbates. When all N_2_ molecules are initially located in top coordination, they change their coordination to bridge during the geometry optimization.

The adsorption complex of one N_2_ ligand with top coordination to the CeO_2_(110) surface model (Figure 4) has a BE of −0.30 eV, and the calculated wavenumber, 2261 cm^−1^, is higher by 9 cm^−1^ than the corresponding value for the gas phase ^15^N_2_ molecule.

### 2.4. Acidity of Hydroxyl Groups

The low-temperature adsorption of CO and N_2_ was also used to determine the acidity of hydroxyl groups on ceria. In order to obtain high-quality spectra, we examined deuterated samples because the noise level is much lower in the ν(OD) region as compared to ν(OH). Acidity was assessed based on the red shift of the ν(OD) modes induced by the probe molecules [25,26]. The greater the shift, the higher the acidity. It should be noted that the CO-induced shift is approximately 2.5 times larger than that induced by N_2_ due to its higher basicity [26,40]. For these experiments, we studied CeO_2_-NC evacuated at 473 and 773 K, respectively.

Generally, the OH/OD groups on ceria are not expected to exhibit high acidity [8,49]. For CeO_2_-NC, two main OD bands were observed around 2745 and 2705 cm^−1^, attributed to the Ce^4+^-associated terminal and bridging OD groups, respectively. Additionally, at lower frequencies, a series of weak bands characteristic of hydrogen-bonded OD groups in cerium oxyhydroxide were detected. It should be noted that cooling the sample to 100 K causes a slight blue shift of the OD bands.

**Figure 5:**
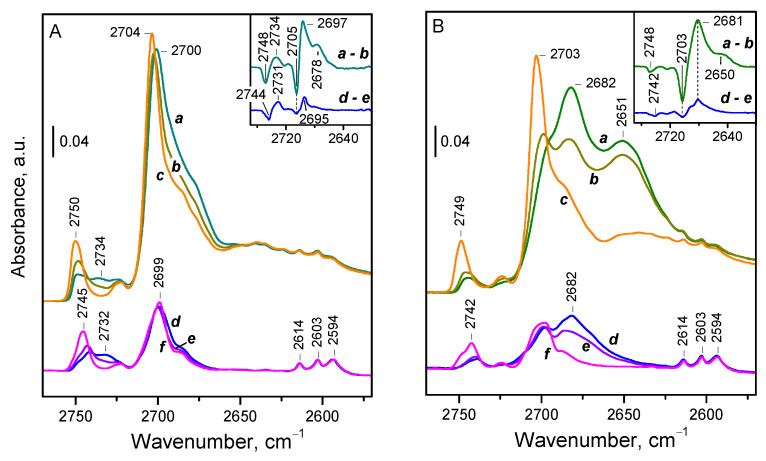
FTIR spectra in the OD region, registered after adsorption of ^15^N_2_ (**A**) and CO (**B**) on CeO_2_-NC pre-evacuated at 473 K (upper spectra a–c) and 773 K (lower spectra d–f): spectra taken immediately after adsorption (a, d), after a certain time (b, e), and after short evacuation (c, f).

Figure 5 demonstrates the effect of CO and N_2_ adsorption on the OD spectra of CeO_2_-NC.

CO adsorption on the sample evacuated at 773 K (Figure 5B, spectra d–f) results in a shift of the bands of terminal and bridging deuteroxyls to a single band around 2682 cm^−1^. Thus, the observed values of Δν(OD) are −61 and −22 cm^−1^, corresponding to −83 and −30 cm^−1^ for Δν(OH), respectively. For comparison, the shift of the SiOH modes on silica is −90 cm^−1^ [26]. Consistent with expectations, the measured acidity for bridging hydroxyls is quite low, lower than the reported value for silanols on silica. However, the shift for terminal OD groups is close to that for silanols, pointing out relatively higher acidity. We explain this discrepancy by the assumption that terminal OD groups adopt a bridging configuration upon complexation with CO [34]. As a result, the CO-induced shift of the bands attributed to terminal OD groups consists of two components: one due to structural transformation and the other due to complexation with CO. Therefore, the actual acidity of terminal OH/OD groups is lower than the measured one. A similar process has been proposed previously for silanols on amorphous silica-alumina [50].

It should also be noted that deuterated hydroxyls associated with cerium oxyhydroxide impurities (bands at 2614, 2603, and 2594 cm^−1^) are not affected by CO, as these groups are already involved in a D-bonding interaction, which is stronger than their interaction with CO.

The changes in OD spectra following adsorbed dinitrogen show that both bands, of the terminal and bridging OD groups, shift very slightly, ~10 cm^−1^, to two new bands (Figure 5A, lower set of spectra d–f). This corresponds to a CO-induced shift of approximately 25 cm^−1^, similar to that observed with CO for bridging deuteroxyls. However, for terminal OD groups, we measured with CO a significantly larger shift of around 80 cm^−1^, explained by the change in the OD binding mode, i.e., their transformation into bridging OD groups. Evidently, ^15^N_2_, being a weaker base, cannot induce similar transformation, thus allowing terminal groups to retain their initial configuration.

Finally, we examined a sample evacuated at 473 K and exhibiting higher OD coverage. Following CO adsorption, two shifted bands are observed at 2681 and 2650 cm^−1^ (Figure 5B, upper set of spectra a–c). The additional shifted band indicates the presence of hydroxyl groups with enhanced acidity, which disappear at higher evacuation temperatures. A similar effect is observed during dinitrogen adsorption (Figure 5A, upper set of spectra a–c). However, in this case, we clearly observed that terminal groups shift by −10 cm^−1^ to one band, while bridging groups shift to two other bands, by −10 cm^−1^ and −15 cm^−1^, respectively. Hence, we can confidently conclude that the hydroxyls with increased acidity are of the bridging type.

### 2.5. Discussion

The Lewis acidity of ceria develops at the expense of dehydration and the dehydroxylation of the surface. At ambient conditions, the {100} and {110} facets are fully hydroxylated [34,48], while the {111} facet is hydrated [34,51]. During evacuation, even at ambient temperature, the {111} facet is dehydrated, thus leaving exposed 7-coordinated Ce^4+^ cations, which are the weakest Lewis acid sites on stoichiometric ceria. With CO, these sites form carbonyls characterized by a band at 2155–2152 cm^−1^ and easily disappear during pumping, even at 100 K. The low value of ν(CO) and its low stability are consistent with the weak adsorbent-adsorbate interaction. Dinitrogen is a weaker base than CO [35,40] and it appears that it is not able to form complexes with the Ce^4+^ sites from the {111} plane, even at 100 K. In principle, it is possible that some highly symmetric and infrared inactive adducts are formed. However, our DFT calculations do not support such a possibility.

Evacuation at higher temperatures leads first to dehydroxylation of the {110} plane via a recombination of the terminal and triply-bridged OH groups and the appearance of 6-coordinated Ce^4+^ cations with enhanced acidity. We also propose that, under similar conditions, a fraction of 6-coordinated cations with similar electrophilicity, but situated on edges, is formed. With CO, these sites form linear complexes (ca. 2171 cm^−1^ for planes and 2175 cm^−1^ for edges), which are converted, at high coverage, into geminal species. However, with the weaker base ^15^N_2_, these Ce^4+^ sites form exclusively linear species characterized by a band at 2258–2257 cm^−1^, with the higher wavenumber component likely characterizing edge sites. No evidence for geminal species was found.

A further increase in the evacuation temperature above 573 K leads to dehydroxylation of the {100} plane via a recombination of doubly-bridged OH groups. The Ce^4+^ formed is 6-coordinated, but the geometric arrangement favors the formation of bridging complexes. As a result, CO adsorption leads to the appearance of carbonyls bridging two Ce^4+^ sites by their carbon atom. The maximum of the carbonyl band is strongly coverage-dependent and is detected at 2169 cm^−1^, at very low coverage, and at 2162 cm^−1^, at intermediate coverage. Note that, in this case, the interaction is essentially electrostatic, and the adducts formed are very different in nature from the classic bridging carbonyls on metal surfaces where the rehybridization of CO occurs. With ^15^N_2_, these sites also form bridging adducts, absorbing at 2252 cm^−1^, and the band maximum is practically coverage-independent due to the weak induction effect of dinitrogen.

In addition to the Ce^4+^ sites, stoichiometric ceria are characterized by surface OH groups that, under certain conditions, can cover significant fractions of the {100} and {110} planes. Both probe molecules, CO and ^15^N_2_, could be used for the estimation of protonic acidity. In both cases, the bands due to the probe molecule interacting with OH groups are of relatively low intensity and are even weaker in the case of ^15^N_2_ due to its low basicity. Good agreement was found between the acidity of the bridging hydroxyls, as measured by the two probe molecules. However, it appears that, in this case, the use of ^15^N_2_ is advantageous because it allows for measuring the acidity of the terminal OH groups. As we proposed earlier, the failure of CO to measure this acidity is likely due to the fact that it, being a relatively strong base, causes the transformation of linear to bridging OH groups when carbonyl adducts are formed.

For convenience, the observed and calculated frequencies of CO and ^15^N_2_ adsorbed on different surface strictures of ceria are summarized in Table 3.

In summary, the results indicate that dinitrogen shows some similarities and some differences from CO as a probe molecule for testing ceria surfaces. The main disadvantage of ^15^N_2_ seems to be its impossibility to detect Ce^4+^ sites on the {111} ceria face. However, this could also be regarded as an advantage because ^15^N_2_ probes only the strongest Lewis acid sites. Another drawback of dinitrogen is the relatively low intensity of its IR band. However, this could be a problem only when studying materials with a low content of cerium. One could also speculate that the spectral interval of the detected bands is too narrow, but this is compensated by the negligible coverage dependence of the band maxima.

On the contrary, it seems that ^15^N_2_ has many advantages as a probe molecule for Ce-containing materials as compared to CO. First, no reactive adsorption that can modify the surface (reduction, formation of carbonates) occurs. Due to weak basicity, ^15^N_2_ affects the surface very weakly, and, as a result, the bands of adsorbed species are practically coverage-independent. Moreover, no geminal species are formed at high coverage. All this allows for an easy comparison between the results obtained in different laboratories. In contrast to CO, ^15^N_2_ gives reliable results on the acidity of the different OH groups on ceria.

Finally, we would like to note that this study was performed with stoichiometric ceria. However, to efficiently use one probe molecule, it is necessary to know how it interacts with reduced Ce^3+^ sites. Investigations in this respect are now in progress in our laboratory. Generally, the complexes of ^15^N_2_ on reduced ceria are detected at lower frequencies and with lower intensity as compared to the stoichiometric samples.

## 3. Materials and Methods

The ceria samples used in this work were investigated and characterized in our previous study [34]. Ce(NO_3_)_3_.6H_2_O (Fluka, Buchs, Switzerland, 99% purity) and NaOH (Merck, Darmstadt, Germany, 99% purity) were used for the synthesis. The CeO_2_-NC and CeO_2_-NR samples were prepared using the hydrothermal method [19,52]. Briefly, 85 mL of an aqueous solution of 5 g of Ce(NO_3_)_3_.6H_2_O were added to 150 mL of 36 wt. % aqueous NaOH solution with vigorous stirring. Then, the reaction mixture was transferred to an autoclave. CeO_2_-NC and CeO_2_-NR were obtained after aging in the autoclave for 24 h at 453 and 373 K, respectively. The suspensions were then centrifuged, the precipitates thoroughly washed with deionized water, dried at 393 K, and finally calcined in air at 673 K for 2 h. The CeO_2_-NP sample was obtained by calcination of a fraction of the CeO_2_-NC sample at 923 K for 1 h. This is in line with literature reports showing surface reconstruction of ceria nanocubes at high temperatures, finally leading to the formation of {111} facets [44,45].

The IR spectra were recorded with a Thermo Scientific Nicolet 6700 FTIR spectrometer (Madison, WI, USA) using an MCT-A detector. Each spectrum was obtained by the accumulation of 64 scans at a spectral resolution of 2 cm^−1^ and a precision of 0.01 cm^−1^. Sample powders were pressed into self-supporting pellets (ca. 10 mg cm^−2^). The latter were treated in situ in a home-made IR cell connected to a vacuum-adsorption apparatus with a residual pressure below 10^−3^ Pa. The cell allowed measurements between ambient temperature and 100 K.

First, the samples were activated by heating in 100 mbar O_2_ at 773 K for 30 min, followed by 30 min of evacuation at the same temperature. To obtain deuteroxylated ceria, the samples were exposed to D_2_O vapor at room temperature and then evacuated. This procedure was repeated several times. Finally, before the adsorption experiment, the samples were evacuated (dehydrated/dehydroxylated) at a certain elevated temperature for 30 min.

Adsorption of the two probe molecules (^15^N_2_ and CO) was performed successively at 100 K. First, dinitrogen adsorption was conducted at an equilibrium pressure of 10 mbar, followed by evacuation. Then, carbon monoxide was adsorbed at an equilibrium pressure of 5 mbar. To ensure good thermal conductivity, He (2 mbar) was added to the system before the introduction of CO or ^15^N_2_. Before use, ^15^N_2_, CO, and He were additionally purified by passing them through a liquid nitrogen trap.

The adsorption experiments were performed using the following gases and adsorbates: CO (Merck, Darmstadt, Germany, >99.5%), ^15^N_2_ (Sigma-Aldrich, St. Louis, MO, USA, 98 at. %), O_2_ (Messer, Bad Soden, Germany, 99.999%), He (Messer, Bad Soden, Germany, 99.999%), and D_2_O (Cambridge Isotope Laboratories, Inc., Tewksbury, MA, USA, 99.9%).

Theoretical modeling was based on periodic Density Functional Theory (DFT) calculations. We used Vienna Ab initio Simulation Package (VASP) [53,54,55] with PW91 [56] exchange-correlation functional and dispersion correction of D2 type [57]. The projector-augmented wave (PAW) method was used to describe the core-valence electron interactions and the calculations were performed in the Γ point only. We employed a plane wave basis with an energy cut-off of 415 eV. All atomic coordinates were optimized until the atomic forces became less than 2 × 10^−4^ eV/pm.

We employed four models of ceria, in which the distance between the ceria moieties in neighboring cells was at least 1.0 nm:

CeO_2_(111) surface slab model with a step—half of the surface three-layer is deleted. The unit cell consists of 60 Ce and 120 O atoms, with a size of 1.322 × 1.984 nm^2^ (α = 60°; β = γ = 90°).

CeO_2_(110) surface model, consisting of Ce_96_O_192_, as the cell parameters are a = 2.1900 nm, b = 1.5486 nm, c = 2.1678 nm, α = β = γ = 90°.

CeO_2_(100) surface model, consisting of Ce_64_O_128_, as the cell parameters are a = 2.1900 nm, b = 1.0950 nm, c = 2.1581 nm, α = β = γ = 90°. Due to the polarity of the surface, we created an O-terminated surface as half of the oxygen atoms were moved from the top surface to the bottom one.

Ce_40_O_80_ nanoparticle model is situated in a parallelepiped unit cell with the dimensions of 2.2 × 1.9 × 1.9 nm.

The binding energy of N_2_ (BE) to Ce^4+^ cations was calculated as the difference between the energy of the adsorption complex on the one hand, and the energies of the pristine ceria model and the isolated dinitrogen molecule on the other. With this definition, the Ce^4+^-N_2_ interaction is energetically favorable when the values are negative. The calculated frequencies for ^14^N_2_ are scaled by 0.9668, representing the ratio between the experimental Raman frequency, 2331 cm^−1^ [58], and the calculated value of the isolated ^14^N_2_, 2411 cm^−1^. The corresponding frequency values for ^15^N_2_ molecule were obtained using an isotope ratio of 1.035.

## 4. Conclusions

Dinitrogen appears to be a suitable IR probe molecule for testing the surface of ceria and ceria-based materials. To avoid any hindrance to gaseous CO_2_, it is recommended to use the ^15^N_2_ isotopologue. When adsorbed on stoichiometric ceria, ^15^N_2_ does not interact with the weakly acidic Ce^4+^ on the (111) planes, but forms well-defined species with the Ce^4+^ sites from the (110) and (100) planes, which are detected in the IR spectra at 2258–2257 and 2253–2252 cm^−1^, respectively. ^15^N_2_ also provides information about the protonic acidity of the OH/OD groups by shifting their maxima to lower frequencies. Note that CO fails to measure the acidity of the terminal OH groups because it causes a change in their binding mode.

Compared to CO as a probe molecule, N_2_ has some advantages. It does not reduce the Ce^4+^ cations and does not form anionic species (as carbonates formed by CO), which can modify the surface. ^15^N_2_ has a weak induction effect, and the maxima of the bands of adsorbed ^15^N_2_ are practically coverage-independent. Finally, dinitrogen can be used for selective determination of protonic acidity.

## Figures and Tables

**Table 1:** Some basic characteristics of the samples studied.

№	Sample	Particle Shape	Average Particle Size [nm] *	S_BET_ [m^2^ g^−1^]	Pore Volume [cm^3^ g^−1^]	Average Pore Diameter [nm]
1	CeO_2_-NC	cubes	27.2	31	0.17	22
2	CeO_2_-NP	polyhedra	29.2	20	0.19	26
3	CeO_2_-NR	rods	6.3	110	0.46	17

* Average particle size determined according to the Scherrer’s equation.

**Table 2:** Binding energies (in eV), interatomic distances (in pm), and calculated vibrational frequencies (in cm^−1^) of N_2_ molecule adsorbed on Ce^4+^ ions of various ceria-based systems: Ce_40_O_80_ nanoparticle, CeO_2_(100) (with checkered (ch) and linear arrangements of the surface oxygen centers), CeO_2_(110), and CeO_2_(111) surface model with step. The shift with respect to isolated N_2_ molecule, Δν, is also shown.

Structure	BE	d(Ce-N)	d(N-N)	Δd(N-N)	ν(^14^N_2_)	ν(^15^N_2_)	Δν
Ce_40_O_80_ apex top	−0.30	309	111.0	−0.1	2333	2254	2
CeO_2_(100)ch—bridge	−0.45	309	111.0	−0.1	2331	2252	0
CeO_2_(100)—bridge	−0.43	312; 313	111.0	−0.1	2332	2253	1
CeO_2_(110)—top	−0.30	304	111.0	−0.1	2340	2261	9
CeO_2_(111)_edge-top	−0.31	304	110.9	−0.2	2340	2261	9
CeO_2_(111)—top	−0.27 ÷ −0.26	297	111.0	−0.1	2334	2255	3

**Table 3:** Summary of binding energies (in eV) and infrared bands (in cm^−1^) of CO and ^15^N_2_ probe molecules characteristic of different ceria surfaces.

Structure	BE(CO) ^a^	BE(N_2_)	ν(CO)exp	ν(CO)calc ^a^	ν(^15^N_2_)exp	ν(^15^N_2_)calc	ν(^14^N_2_)exp. ^b^
Apex—top	−0.39	−0.30	2175	2183	2258	2254	2337
Edge (111)—top	−0.40	−0.31	2175	2180	2258	2261	2337
Ce(100)—bridge	−0.62	−0.43	2162	2155	2253	2253	2332
Ce(110)—top	−0.37	−0.30	2171	2165	2257	2261	2336
Ce(111)—top	−0.38	−0.27	2155	2149	-	2255	-

^a^ Data from Ref. [34]. ^b^ Calculated on the basis of the experimental values for ν(^15^N_2_).

## Data Availability

All data generated during this study are provided in the manuscript and in the Supporting information.

## Supplementary Materials

Supplementary data to this article can be found online at https://www.mdpi.com/article/10.3390/molecules29153608/s1. Figure S1: IR spectra of ^15^N_2_ (different coverages) adsorbed at 100 K on CeO_2_-NC, pre-evacuated at 773 K; Figure S2: Adsorption complexes of N_2_ with Ce^4+^ cations on the CeO_2_(111) model; Figure S3: Electron density difference between the linear and bridge coordination of N_2_ on the (100) CeO_2_ surface.
