# Supplementary material for: N2 as an Efficient IR Probe Molecule for the Investigation of Ceria-Containing Materials
Source: Molecules. 2024 Jul 30;29(15):3608. doi: 10.3390/molecules29153608 (PMC11314509; doi:10.3390/molecules29153608)
Supplement: Supplementary file 1 [file molecules-29-03608-s001.zip › molecules-3111664-supplementary.pdf]

SUPPORTING INFORMATION

N_2 as an Efficient IR Probe Molecule for Investigation of Ceria-containing Materials

Kristina K. Chakarova,[1] Mihail Y. Mihaylov[1], Konstantin I. Hadjiivanov[1], Bayan Karapenchev,[1,2] Iskra Z. Koleva,[2] Georgi N. Vayssilov,[2] Hristiyan A. Aleksandrov[1,2]

[1] Institute of General and Inorganic Chemistry, Bulgarian Academy of Sciences, Sofia 1113, Bulgaria

[2] Faculty of Chemistry and Pharmacy, University of Sofia, 1126 Sofia, Bulgaria

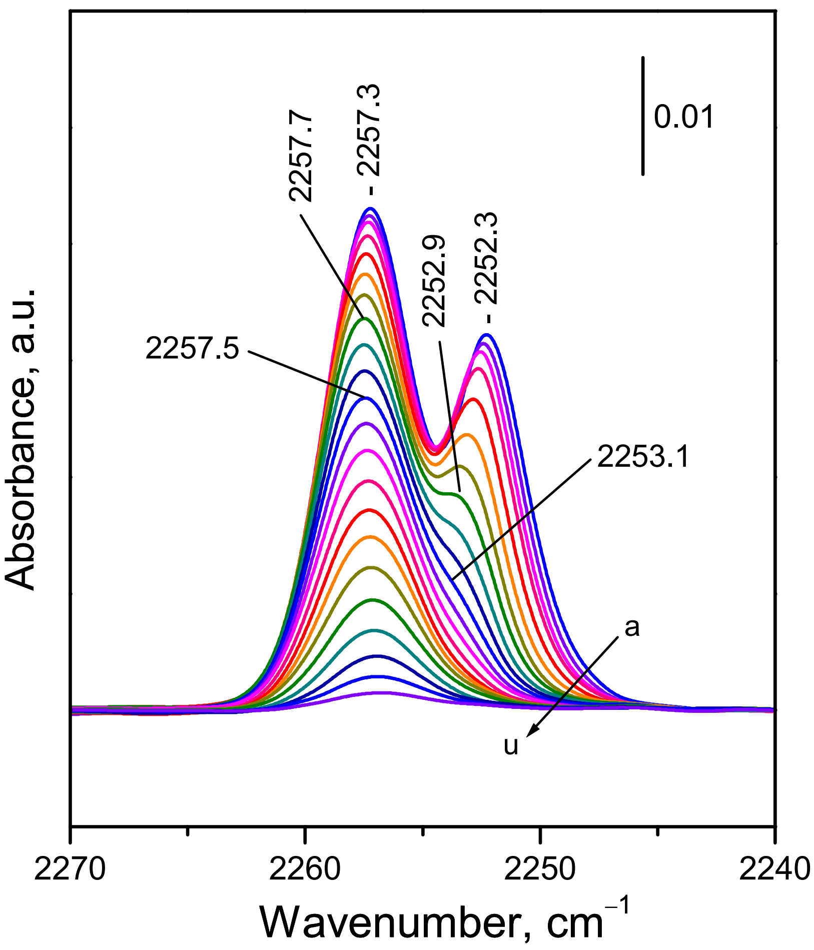

Figure S1. IR spectra of $^{15}N_2$ adsorbed at 100 K on CeO_2-NC, pre-evacuated at 773 K. Equilibrium $^{15}N_2$ pressure of 0.5 (a) and 0.1 mbar (b) and development of the spectra in the conditions of dynamic vacuum (c-u). The band maxima are determined according to the second derivatives of the spectra.

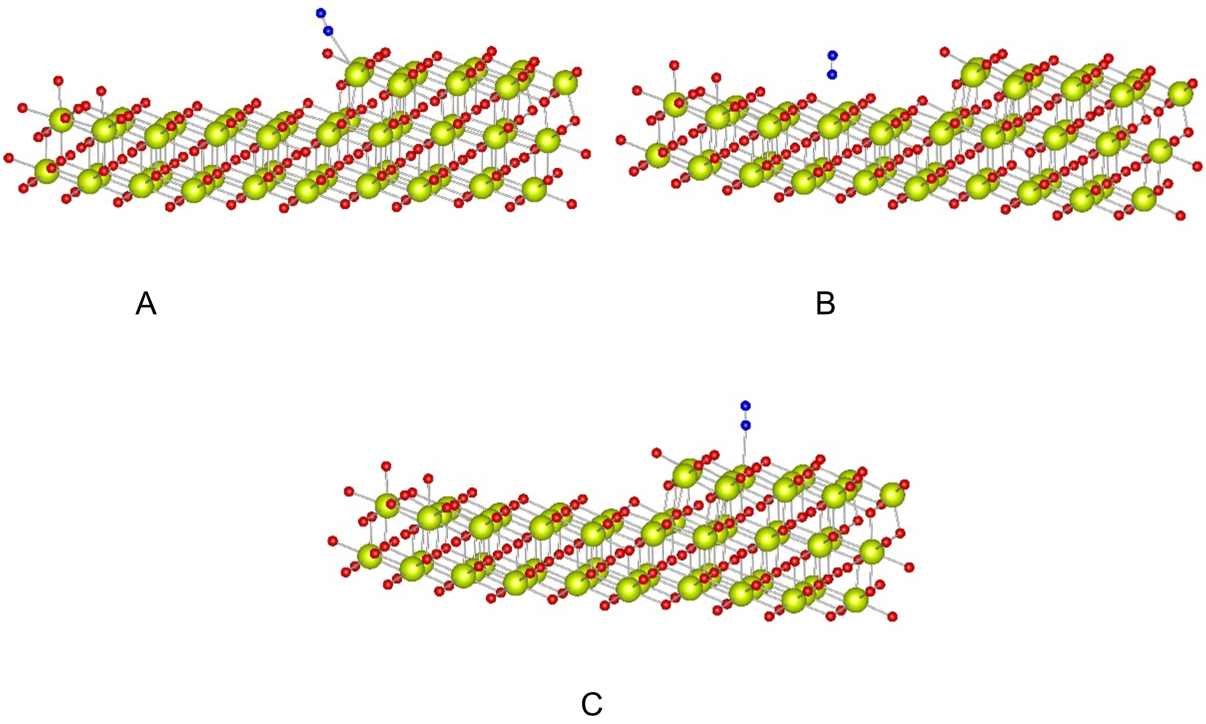

Figure S2. Adsorption complexes of N_2 with Ce^{4+} cations on the $CeO_2(111)$ model with a step between the {111} facets. A. Adsorption on the step. B and C. Adsorption on the (111) plane.

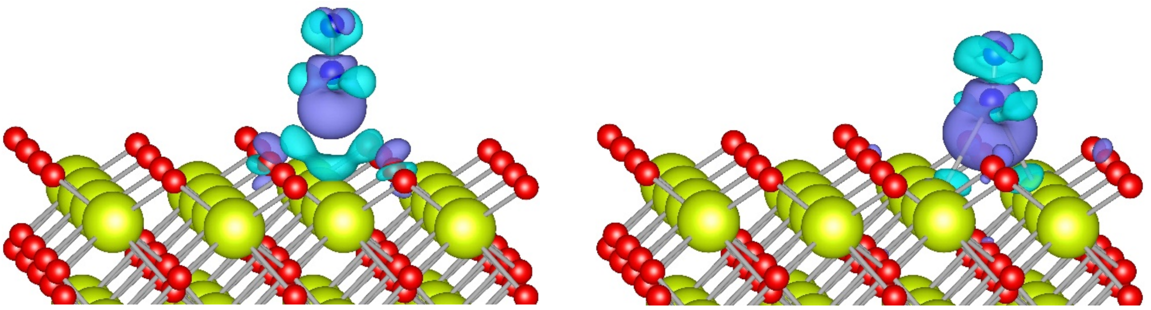

Figure S3. Electron density difference between the linear (left panel) and bridge (right panel) coordination of N_2 on the (100) CeO_2 surface with respect to the electron density of (100) CeO_2 surface and isolated N_2 molecule. Dark blue areas correspond to increase of the electron density in the $N_2/CeO_2(100)$ system, while cyan areas correspond to electron density depletion.
